# Administration of a Multi-Strain Probiotic Product to Women in the Perinatal Period Differentially Affects the Breast Milk Cytokine Profile and May Have Beneficial Effects on Neonatal Gastrointestinal Functional Symptoms. A Randomized Clinical Trial

**DOI:** 10.3390/nu8110677

**Published:** 2016-10-27

**Authors:** Maria Elisabetta Baldassarre, Antonio Di Mauro, Paola Mastromarino, Margherita Fanelli, Domenico Martinelli, Flavia Urbano, Daniela Capobianco, Nicola Laforgia

**Affiliations:** 1 Department of Biomedical Science and Human Oncology, Neonatology and Neonatal Intensive Care Unit, “Aldo Moro” University of Bari, Bari 70100, Italy; antonio.dimauro@uniba.it (A.D.M.); domenico_tine@libero.it (D.M.); flaviaurbano@libero.it (F.U.); nicola.laforgia@uniba.it (N.L.); 2 Department of Public Health and Infectious Disease, “Sapienza” University of Rome, Rome 00100, Italy; paola.mastromarino@uniroma1.it (P.M.); daniela.capobianco@uniroma1.it (D.C.); 3 Department of Interdisciplinary Medicine, “Aldo Moro” University of Bari, Bari 70100, Italy; margherita.fanelli@uniba.it

**Keywords:** pediatric gastroenterology, probiotics/prebiotics, immunology, functional GI diseases

## Abstract

Background: Probiotic supplementation to women during pregnancy and lactation can modulate breast milk composition, with immune benefits being transferred to their infants. Aim: The aim of the study was to evaluate the effect of high-dose probiotic supplementation to women during late pregnancy and lactation on cytokine profile and secretory IgA (sIgA) in breast milk and thus to study if differences in breast milk composition can affect lactoferrin and sIgA levels in stool samples of newborns. The safety of maternal probiotic administration on neonatal growth pattern and gastrointestinal symptoms were also evaluated. Methods: In a double-blind, placebo-controlled, randomized trial, 66 women took either the probiotic (*n* = 33) or a placebo (*n* = 33) daily. Levels of interleukins (IL-6, IL-10 and IL-1β), transforming growth factor-β1 (TGF-β1), and sIgA in breast milk; and the level of sIgA and lactoferrin in newborn stool samples were analyzed at birth and then again at one month of life. Antropometrical evaluation and analysis of gastrointestinal events in newborns was also performed. Results: Probiotic maternal consumption had a significant impact on IL6 mean values in colostrum and on IL10 and TGF-β1 mean values in mature breast milk. Fecal sIgA mean values were higher in newborns whose mothers took the probiotic product than in the control group. Probiotic maternal supplementation seems to decrease incidence of infantile colic and regurgitation in infants. Conclusion: High-dose multi-strain probiotic administration to women during pregnancy influences breast milk cytokines pattern and sIgA production in newborns, and seems to improve gastrointestinal functional symptoms in infants.

## 1. Introduction

Several papers have shown that probiotic supplementation to women during pregnancy and lactation can modulate the microbial milk composition and the level of different breast milk immunity and immunity-modulating molecules, with health benefits ranging from gastrointestinal symptoms to allergies, transferred to the children [1].

Our previously published studies demonstrated that the same high concentration multi-strain probiotic, when supplemented to mothers in the perinatal period, was associated with a modulation of the vaginal microbiota and vaginal cytokine secretion [2] and with an increase in levels of breast milk bifidobacteria and lactobacilli in women with vaginal deliveries, regardless of the milk concentrations of functional components such as oligosaccharides and lactoferrin [3].

An improved microbial milk composition might have important consequences for the health of the newborn [4]. We did not, however, find any correlation between the amount of bifidobacteria and lactobacilli in maternal breast milk and levels in the feces of infants [5].

We have speculated that cytokines and other immunoregulatory factors, modulated by probiotic supplementation, might have a key role in the establishment of the microbiota of human milk. 

The primary outcomes of this study were to evaluate if maternal probiotics supplementation can modulate the cytokine profile and secretory IgA (sIgA) in breast milk and thus to study if differences in breast milk composition can affect lactoferrin and sIgA levels in newborn stool samples.

Our secondary outcome was to evaluate the safety of high-concentration administration of maternal probiotics for newborns, evaluating growth and the onset of the most frequent gastrointestinal symptoms in infants. It has been hypothesized that perinatal manipulation of gut microbiota by probiotics supplementation can result in a decrease of gastrointestinal functional symptoms [6].

A previous study demonstrated that early life probiotic administration could prevent the onset of gastrointestinal functional symptoms in newborns [7]. The correct mechanism of action of probiotics in this field has not thus far been clarified, but it appears to be mediated by activity on colonic intrinsic sensory neurons with an improvement in gut motility, as well as positive effects on function and visceral pain [8]. Recently, a study on rats allowed the identification of novel regulatory mechanisms and specific patterns of genic expression caused by exposure to the same high concentration multi-strain probiotic which may have clinical readouts in visceral pain [9]. In addition, it is important to note that health benefits conferred by probiotics bacteria are strain-specific.

## 2. Experimental Section

### 2.1. Study Design and Patients

This was a double-blind, randomized, placebo-controlled clinical trial (clinicaltrials.gov: NCT01367470), performed to assess the effect of maternal probiotic administration on the breast milk cytokine pattern and sIgA production.

Patients and methods are described in detail in our previous paper where we evaluated the effect of oral probiotic supplementation to women on breast milk levels of beneficial bacteria [4].

Healthy, pregnant women, aged 18–44 years, who had been admitted with a low obstetric risk at the Unit of Obstetrics and Gynecology, Department of Biomedical and Human Oncological Science (DIMO), University of Bari, Bari, Italy between April 2011 and December 2013, were enrolled.

Exclusion criteria were: (a) pre-existing clinical conditions such as diabetes, hypertension, autoimmune diseases, asthma, allergies, renal or hepatic diseases, viral, bacterial or protozoan infection, anemia (hemoglobin, Hb < 10 g/L); (b) twin pregnancies; (c) pregnancy disease and preterm deliveries; (d) smoking more than 10 cigarettes per day; (e) use of other probiotics during the study protocol.

Informed consent was obtained at the enrolment, in accordance with the local Ethics Committee, (University of Bari, Policlinico Hospital of Bari, Medical School, BARI, Italy) which reviewed and approved the study protocol. 

All women enrolled were randomized to receive either oral probiotics or a placebo supplementation daily, four weeks before the expected delivery date (36th week of pregnancy) until four weeks after delivery. Randomization was performed using a computer-generated allocation sequence. All participants, as well as scientific and medical personnel dedicated to the study and distributing the study agents or assessing the samples and analyses, were blinded to group assignment.

A high-concentration multi-strain probiotic supplement was used, consisting of packets containing 900 billion viable lyophilized bacteria of four different strains of lactobacilli (*L. paracasei* DSM 24733, *L. plantarum* DSM 24730, *L. acidophilus* DSM 24735, and *L. delbrueckii* subsp. *bulgaricus* DSM 24734), three strains of bifidobacteria (*B. longum* DSM 24736, *B. breve* DSM 24732, and *B. infantis* DSM 24737), and one strain of *Streptococcus thermophilus* DSM 24731, produced at Danisco-Dupont, WI, USA and currently sold in Continental Europe and USA under the brand Vivomixx^®^ and Visbiome^®^, respectively. 

The placebo was composed of corn starch and was identical in sensory properties in order to maintain a double-blind status.

A nutritionist gave dietary guidance to each mother, according to the collected anthropometric values and a dietary interview at the end of the first trimester of pregnancy. No specific dietary restrictions during pregnancy and lactation were recommended, with the exception of other commercial products containing probiotics. 

Mothers who delivered by cesarean section received an “intra-partum” single dose of 2 g of a “third generation” cephalosporin. 

Maternal and neonatal characteristics were collected at baseline. 

### 2.2. Analysis of Breast Milk

To evaluate if maternal probiotic supplementation modulated the cytokine profile and sIgA in breast milk, two samples of maternal milk were collected by manual extraction: colostrum within 72 h after delivery (T0) and mature milk at 30 days postpartum (T30). 

Levels of interleukins (IL-6, IL-10 and IL-1β) and transforming growth factor-β1 (TGF-β1) were analyzed using commercial enzyme-linked immunosorbent assay (ELISA) kits (CLB Pelikine Compact, Research Diagnostics Inc., Flandern, NJ, USA, and R&D Systems Inc., Minneapolis, MN, USA, respectively) according to the manufacturer’s recommendations. All samples were analyzed in duplicate. Values are expressed in pg/mL. IgA levels were determined by nephelometry (Behring Nephelometer Analyzer; Dade-Behring, Marburg, Germany). 

### 2.3. Analysis of Infant Stool Samples

To evaluate if maternal probiotic supplementation modulated lactoferrin and sIgA concentration in the stool samples of newborns, two stool samples were collected at T0 and T30. All samples were collected from diapers in sterile plastic tubes and stored at −80 °C until analysis. Lactoferrin concentration in newborn stool samples was measured by ELISA using the Human Lactoferrin ELISA kit (Bethyl Laboratories, Inc., Montgomery, TX, USA).

IgA levels were determined by nephelometry (Behring Nephelometer Analyzer; Dade-Behring, Marburg, Germany).

### 2.4. Safety Evaluation

To assess the safety of maternal probiotic supplementation on newborn growth patterns, anthropometric data (weight and height) were collected at T0 and T30. Growth patterns were assessed through body mass index analysis. Failure to thrive was considered when an infant’s weight was less than 80% of the ideal weight for its age [10]. 

According to the study protocol, a structured diary on gastrointestinal events in newborns, such as number of minutes of inconsolable crying per day (infantile colic as already described in literature [11]), number of regurgitation episodes per day, number of bowel movements per day and stool consistency, according to the Bristol Stool Form Scale for children was given to parents [12].

We identified infant regurgitation and infant colic following the Rome III criteria in the neonatal/toddler period [13]. We defined colic in infants as otherwise healthy infants who displayed paroxysms of irritability, fussing or crying which started and stopped without an obvious cause in episodes lasting three or more hours per day and occurring at least three days per week for at least three weeks during the study period. We defined infants with functional regurgitation as infants who regurgitated two or more times per day for three or more weeks during the study period, in absence of retching, hematemesis, aspiration, apnea, failure to thrive, abnormal posturing or who had feeding or swallowing difficulties during that time.

### 2.5. Statistical Analysis

The sample size was calculated using power Statistical Analysis Systems statistical software package version 9.3 (SAS Institute, Cary, NC, USA) based on the effect of the administration of a probiotic product on breast milk TGF-β1 level [14]. The level of significance was set to 5% (two-sided) and the power to 80%. We calculated that a minimum sample size of 54 mothers was required to detect a difference in the breast milk TGF-β1 level between treatment and the placebo group. Maternal and newborn quantitative data were expressed as mean, SD and range. The t test for unpaired samples was used to compare the intervention groups.

The Chi-square test and the Fisher exact test were used to compare the qualitative aspects in the two groups.

A two-way repeated measure ANOVA was performed to evaluate the time effect, the treatment effect and the interaction effect on the cytokine profile and sIgA values in breast milk as well as on faecal lactoferrin and sIgA values. 

When data was not normally distributed according to Shapiro-Wilks, logarithmic transformation was performed.

The Chi-square test and Relative Risk (RR) were computed to evaluate the possible link between probiotic supplementation and functional gastrointestinal disorder (FGID) onset. 

Logistic regression was performed to assess which factors (probiotics, type of delivery or maternal milk/both maternal-bottle milk) had the greatest impact on FGID onset. 

## 3. Results

**Figure 1:**
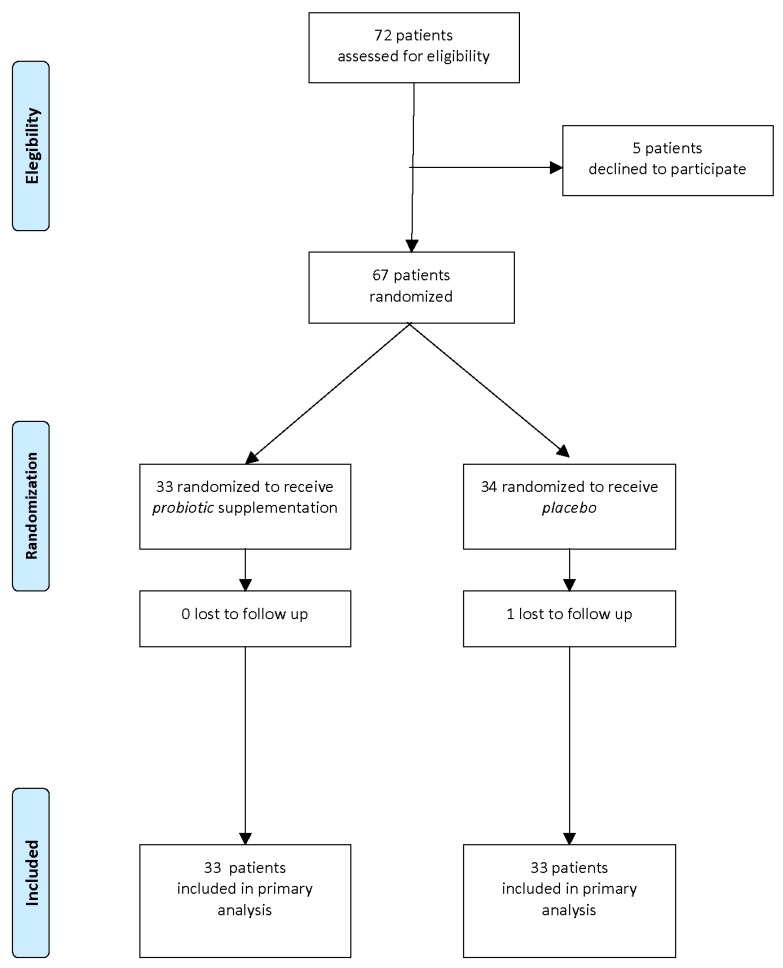
Study flow diagram.

A flow diagram of the progress through the phases of this parallel randomized trial of two groups is shown in Figure 1. 

Of the 72 mother/newborn pairs assessed for eligibility, 67 were enrolled and randomized to receive probiotics (T: probiotic, *n* = 33) or placebo (C: placebo, *n* = 34). 

After one month of intervention, 1 pair was lost to follow-up and 66 pairs completed the study and were entered in the analysis of results.

Characteristics of mothers and newborns at baseline are detailed in Table 1.

No significant differences regarding maternal age, Body Mass Index (BMI) in first trimester, weight gain during pregnancy, use of antibiotics in perinatal periods, or newborn characteristics (gestational age, birth weight, sex, type of delivery and type of feeding) were observed between the two groups at baseline thus showing the randomization procedure was successful. 

No detectable side effects were observed in mothers or in newborns.

**Figure 2:**
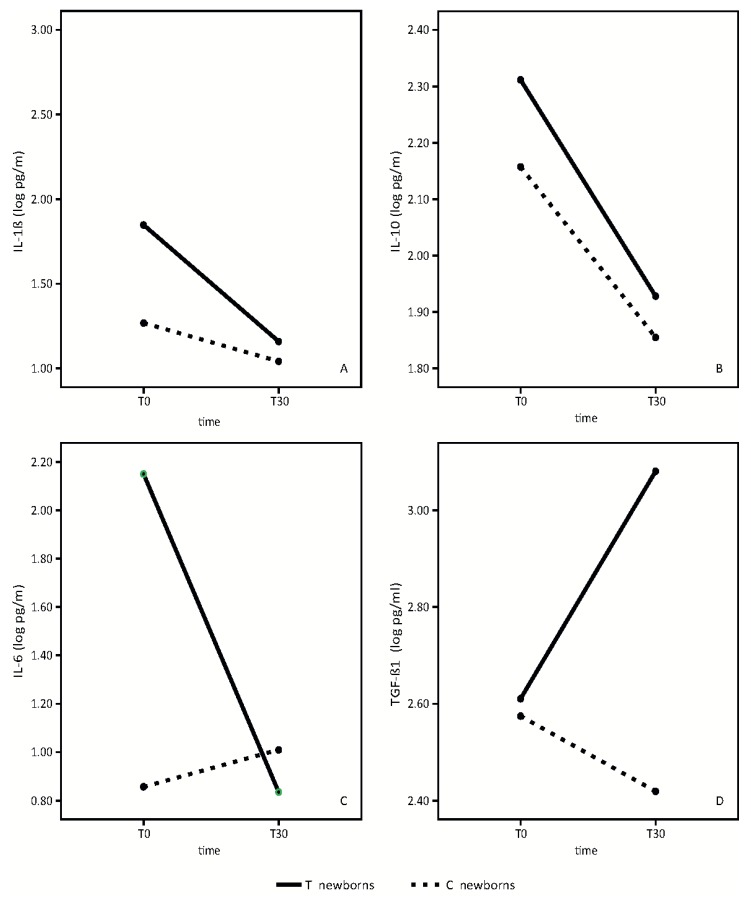
Breast milk cytokine patterns in treatment (T) and control (C) newborns at observational times T0 and T30. (**A**) (Interleukin-1β mean values); (**B**) (Inteleukin-10 mean values); (**C**) (Interleukin-6 mean values); (**D**) (Transforming Growth Factor mean values).

**Figure 3:**
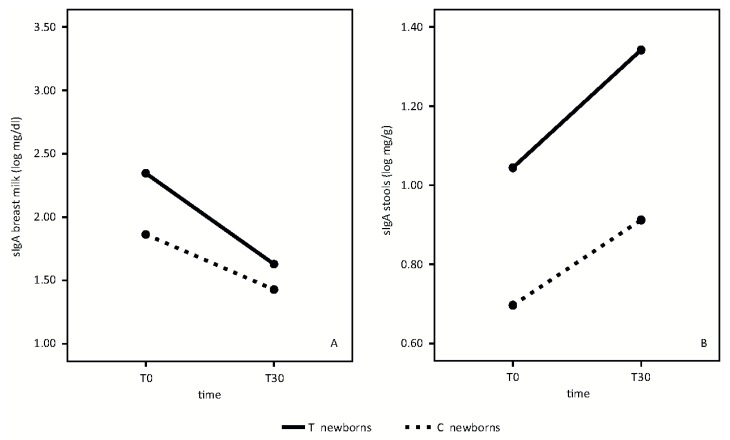
Secretory IgA (sIgA) in breast milk and stools, in treatment (T) and control (C) groups at observational times T0 and T30. (**A**) (Secretory IgA (sIgA) mean values in breast milk); (**B**) (Secretory IgA (sIgA) mean values in stools).

**Figure 4:**
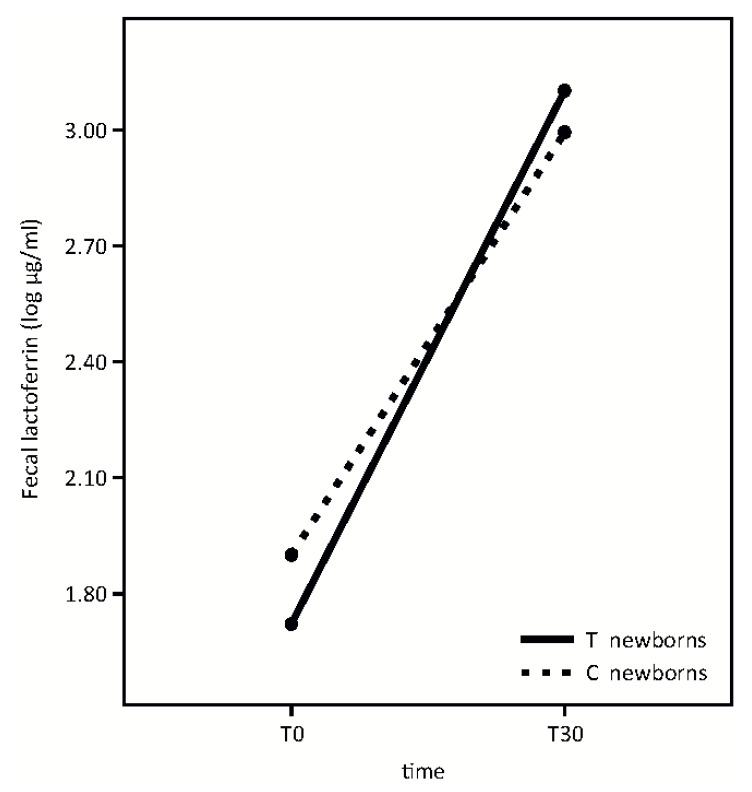
Fecal lactoferrin in treatment (T) and control (C) groups at observational times T0 and T30.

Primary outcomes at T30 follow-up are shown in Figure 2, Figure 3 and Figure 4. 

**Figure 5:**
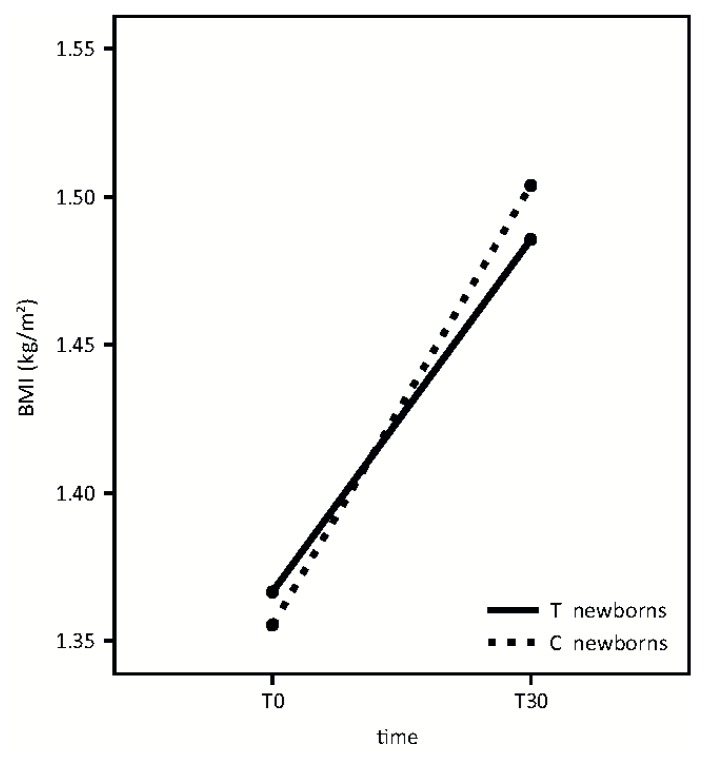
Growth pattern in treatment (T) and control (C) groups.

**Figure 6:**
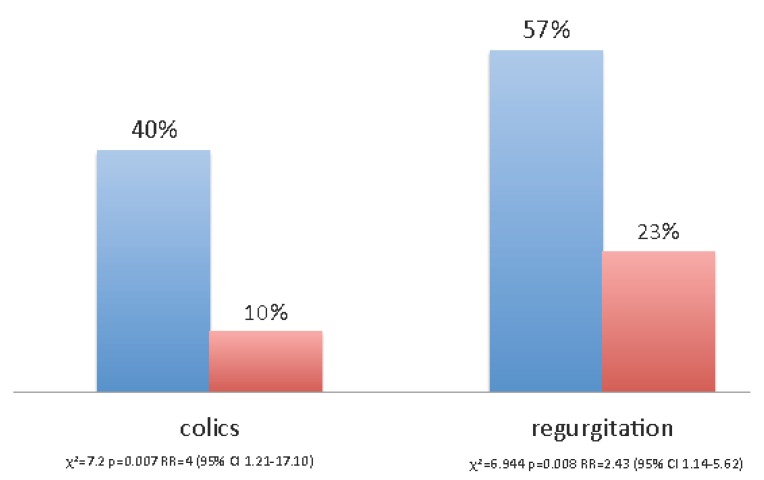
Functional gastrointestinal disorders (FGIDs) in treatment (T) and control (C) groups.

Secondary outcomes are shown in Figure 5 and Figure 6.

### 3.1. Breast Milk Cytokine Analysis

Results of the analysis of the breast milk cytokine pattern of both the placebo and probiotic-supplemented women are shown in Figure 2.

We did not find any statistically significant differences in IL-1β between the placebo group and the group of probiotic-supplemented mothers. (Figure 2A). In both groups, we observed a slight reduction of IL-1β over time (time effect: F = 4.41, *p* = 0.05; treatment effect: F = 1.28, *p* = 0.28, interaction effect: F = 1.12, *p* = 0.31).

IL10 mean values are significantly higher in the breast milk of probiotics supplemented mothers in respect to control mothers (Figure 2B). IL10 values decreased in both groups from T0 to T30 (time effect: F = 145.72, *p* < 0.001; treatment effect: F = 6.63, *p* = 0.02, interaction effect: F = 2.04, *p* = 0.17).

IL6 mean values in colostrum were significantly different between the two groups. (Figure 2C). IL-6 concentrations were significantly higher in colostrum of mothers receiving the probiotics. In probiotic-supplemented mothers, IL-6 values decreased from T0 to T30, reaching approximately the same values found in the mature milk of the control mothers. (Time effect: 48.13, *p* < 0.001; treatment effect: F = 24.35, *p* < 0.001, interaction effect: F = 76.82, *p* < 0.001). No significant variation in control milk values were observed.

TGF-β1 mean values were significantly higher in the mature breast milk of supplemented mothers in respect to control breast milk (Figure 2D).

TGF-β1 mean values increased significantly in the supplemented group from T0 to T30, while the values decreased in the control group (time effect: F = 5.21, *p* = 0.04; treatment effect: F = 11.76, *p* = 0.004, interaction effect: F = 20.63, *p* < 0.001).

Logistic regression analysis showed that the only factor with a significant impact on breast milk cytokines was maternal probiotic consumption. No difference was found based on mode of delivery (data not shown).

### 3.2. sIgA Analysis

sIgA analyses in breast milk and newborn stool samples are shown in Figure 3.

In breast milk, sIgA mean values decreased from T0 to T30 in both groups, without any statistically significant differences (Figure 3A) (time effect: F = 22.69, *p* < 0.001; treatment effect F = 3.57, *p* = 0.08, interaction effect F = 1.35, *p* = 0.26).

In newborn stools, sIgA increased from T0 to T30 in both groups. Mean values were higher in newborns whose mothers took the probiotic product than in the control group (Figure 3B) (Time effect: F = 15, *p* = 0.02; treatment effect: F = 10.62, *p* = 0.005, interaction effect: F = 15, *p* = 0.69).

### 3.3. Fecal Lactoferrin Analysis

Fecal lactoferrin analysis is shown in Figure 4.

Fecal lactoferrin concentration significantly increased from T0 to T30 in both groups with no significant differences in newborns whose mothers took the probiotic product in respect to the control group (Time effect: F = 54.76, *p* < 0.001; *p* = 0.39; treatment effect: F = 0.03, *p* = 0.87; interaction effect: F = 0.73).

### 3.4. Safety, Growth Pattern and Infant FGIDs

The probiotic supplementation was well tolerated in both the mothers and the newborns and no side effects were reported.

A similar growth pattern was detected in the two groups, according to BMI (Figure 5). (Time effect: F = 118.95, *p* < 0.001; treatment effect: F = 0.01, *p* = 0.92; interaction effect: F = 1.43, *p* = 0.24). 

IL-6 concentration in breast milk in the first months of life could play a pivotal role in the accrual of fat and lean body mass. We found a non-statistically significant, downward trend, between IL-6 milk concentration and infant BMI, at T30.

Colic and regurgitation were more frequent in the placebo group than in the probiotic group (Figure 6) (colics: *χ*^2^ = 7.2, *p* = 0.007; Relative Risk = 4 (95% C.I.: 1.21–17.10); regurgitation: *χ*^2^ = 6.944, *p* = 0.008; Relative Risk = 2.43 (95% C.I.: 1.14–5.62). Logistic regression analysis showed that the only factor with a significant impact on colic and regurgitation was maternal probiotic consumption (data not shown). Newborns with lower frequency of regurgitation had received milk with higher TGF-β1 concentrations compared to the newborns with more frequent regurgitation. (1432.30 vs. 265.57 pg/mL, *t* = 3.91; *p* = 0.004).

No significant differences in number of bowel movements (3.7 vs. 4.2, *t* = 1.17, *p* = 0.246) and consistency of stools (*χ*^2^ = 3.53, *p* = 0.317) were found between the two groups.

## 4. Discussion

In this study, we evaluated the mean values in colostrum and mature milk of two anti-inflammatory cytokines, TGF-β1 and IL-10, and two pro-inflammatory cytokines, IL-1β and IL-6. 

Our data shows a significant temporal increase of breast milk TGF-β1 in probiotic-supplemented mothers with respect to the control group (Figure 2D). Furthermore, the mean value of IL-10 in both colostrum and mature milk was higher in supplemented mothers than in the control group (Figure 2B). The beneficial effect of this probiotic preparation on the anti-inflammatory cytokine profiles of breast milk was not influenced by the mode of delivery but only by the probiotic administration to mothers.

During early infancy, TGF-β1 probably has a crucial effect on the development of the immature gastrointestinal tract by influencing IgA production and oral tolerance induction [15]. On the basis of the known role of TGF-β1 on the level of sIgA [16], that serves as a first line of defense in gut mucosal immunity [17], it is possible to hypothesize that the increase of TGF-β1 levels in breast milk, induced by this high dose probiotic preparation, may be responsible for the significantly higher sIgA levels found in stools of infants whose mothers took the probiotic product at T30 (Figure 3B). 

As the TGF-β1 breast milk levels at T0 are not different between the two groups (Figure 2D), the higher sIgA concentrations at T0 in the supplemented-group newborn stool samples (Figure 3B) could be an early expression of the mucosal immune system in the developing fetus. Amniotic fluid contains levels of IgA, of fetal origin, supporting the hypothesis that newborns enter the world having first been bathed in an environment created by the mother, and in our experience, influenced by our probiotic supplementation [18].

Moreover, the mean value of sIgA in breast milk is higher in treated mothers, although it did not quite reach statistical significance (Figure 3A).

A recent paper demonstrates how cytokines exert differential effects on the muscarinic receptor on the intestinal longitudinal smooth muscle and explains the basis for altered gut motility and muscle contractility by intestinal inflammation. In this scenario, TGF-β1 seems to enhance contractility on intestinal longitudinal smooth muscle, suggesting anti-regurgitation effects [19].

In line with these results, our data shows that the newborns with a lower frequency of regurgitation had received milk with higher TGF-β1 concentrations, compared to the newborns with a higher frequency of regurgitation.

IL-10 plays a pivotal role in the maintenance of homeostasis and it has been well known for many years that elevated IL-10 levels suppress inflammation [20]. Our data shows higher mean values of IL-10 in both colostrum and mature milk in probiotic-supplemented mothers compared to the control group (Figure 2D). Other studies demonstrated the efficacy of certain bacteria strains, selected for their ability to increase cytokine IL-10 levels, in the prophylaxis and/or treatment of colic [5].

We can speculate that the increased level of IL-10 in maternal breast milk, induced by our probiotic preparation, may have been responsible for the reduction of colic symptoms in the infants.

In the evaluation of the pro-inflammatory cytokine profile, our data shows a significant temporal decrease of IL-6 in breast milk in probiotic-supplemented mothers, although the mean value in colostrum was significantly higher in probiotic-supplemented mothers compared to the control group. In the mature milk, IL-6 levels reached approximately the same values of the control group. (Figure 2C). 

A previous pilot study showed that IL-6 concentration in breast milk in the first months of life could play a pivotal role in the accrual of fat and lean body mass [21]. According to the authors, IL-6 concentration in breast milk had pervasive negative associations with infant growth and adiposity. Our data does not provide conclusive evidence of a correlation between IL-6 concentration in breast milk and infant BMI, although a downward trend, not statistically significant, can be observed at T30. Further studies with a longer follow-up are necessary to better clarify this hypothesis.

With respect to the IL-1β mean value, we found a slight reduction in value between colostrum and mature milk, with no statistically significant differences between probiotic supplemented mothers and the control group (Figure 2A).

Finally, our results indicate that the administered probiotic microorganisms do not affect fecal lactoferrin concentration at the time points studied (Figure 4). According to our previous evidence [22], lactoferrin fecal concentration is extremely high in infants and significantly increases from T0 to T30. This growth occurs while breast milk lactoferrin concentration decreases longitudinally [5].

Our results finally indicate that oral probiotics supplementation to mothers in the perinatal period may also have consequences on inconsolable crying and regurgitation in the first month of life, however this data cannot be considered conclusive because the sample size calculation power was not adequate to evaluate differences between groups. 

In our previous study, we did not observe any significant differences in newborn gut microbiota composition between the placebo and the control groups, suggesting that maternal probiotic supplementation does not modify the amount of intestinal beneficial bacteria in the newborn. This also supports the hypothesis that colonization of the gut may not be essential for probiotic biological effects.

The beneficial effect of maternal probiotic supplementation on neonatal gastrointestinal discomfort appears to be dependent on differences in the cytokine profile in breast milk.

## 5. Conclusions

Our study demonstrates that maternal supplementation with this specific high-concentration probiotic preparation modulates cytokines in the mammary gland and a sIgA synthesis in newborn gut mucosa. It has also been demonstrated that this maternal supplementation may decrease the onset of gastrointestinal functional symptoms.

A routine supplementation of high-dose probiotics during pregnancy and lactation cannot be recommended yet, however. Further research in this field is needed to confirm our results and to identify mothers who could have benefits from perinatal supplementation. It has been demonstrated, recently, that a mother with a FGID could predispose her children to FGID [23]. Based on our results it would be interesting to verify if early supplementation of high-dose probiotics could be proposed for mothers suffering from FGID.

## Figures and Tables

**Table 1:** Demographic and clinical characteristics of the study population.

Characteristics	Placebo (C = *n* (33))	Probiotic (T = *n* (33))	*p*
*Mothers*			
Maternal age, mean (SD) [range]	32.4 (5.1) [20–41]	34.2 (4.2) [18–40]	n.s.
Maternal Body Mass Index (BMI) in first trimester, mean (SD), kg/m^2^	21.3 (2.0)	19.8 (5.9)	n.s.
Maternal weight gain during pregnancy, mean (SD), kg	13.7 (5.3)	11.7 (3.6)	n.s.
Use of antibiotics in the perinatal period, *n* (%)	2 (6.0)	3 (9.1)	n.s.
*Newborns*			
Gestational age, mean (SD) [range], week	39.0 (1.1) [37–41]	39.4 (0.95) [37–41]	n.s.
Vaginal delivery, *n* (%)	19 (57.6)	23 (69.7)	n.s.
Elective caesarean delivery (%)	7 (21.2)	6 (18.2)	n.s.
Birth weight, mean (SD) [range], g	3415 (501.2) [2760–4730]	3350 (479.8) [2440–4550]	n.s.
Fed with only maternal milk, *n* (%)	30 (90.9)	29 (87.9)	n.s.
Fed with both maternal milk and formula, *n* (%)	3 (9.1)	4 (12.1)	n.s.

Adapted from reference [5].

## Abbreviations

The following abbreviations are used in this manuscript:

Ig	Immunoglobulin
LAB	Lactic acid bacteria
AC	*Acremonium* cellulase
sIgA	Secretory IgA
IL	Interleukin
TGF-β1	Transforming growth factor-β1
Hb	Haemoglobin
FGIDs	Functional gastrointestinal disorders
BMI	Body Mass Index
